# Evaluation of Changes in Pleural Pressure, Spirometry and Their Effects on Dyspnoea and the Six-Minute Walk Test (6-MWT) Following Thoracentesis for Symptomatic Pleural Effusion

**DOI:** 10.7759/cureus.86286

**Published:** 2025-06-18

**Authors:** Jineesh Joseph, Somenath Kundu, Prasanth Prasad, Aneesa Shahul

**Affiliations:** 1 Pulmonology, Sree Gokulam Medical College and Research Foundation, Trivandrum, IND; 2 Pulmonology, Institute of Post Graduate Medical Education and Research (IPGMER) and Seth Sukhlal Karnani Memorial (SSKM) Hospital, Kolkata, IND; 3 Medicine, Sree Gokulam Medical College and Research Foundation, Trivandrum, IND; 4 Pulmonology, Travancore Medical College, Kollam, IND

**Keywords:** dyspnoea, effort tolerance, large pleural effusion, modified borg scale, pleural pressure, six-minute walk test, unilateral pleural effusion

## Abstract

Introduction

The presence of pleural effusion impairs daily activity, including a decrease in exercise capacity. Aspiration of pleural fluid results in improvement of symptoms, but the impact, especially on exercise tolerance, has not been adequately studied.

Methods

Forty-eight patients with moderate to massive unilateral pleural effusion documented by chest radiograph were included in this prospective hospital-based study. The six-minute walk test (6-MWT), modified Borg dyspnoea scale, forced vital capacity (FVC), and forced expiratory volume in the first second (FEV1) were analysed before and 24 hours after the removal of pleural fluid. Pleural pressure was monitored during aspiration using over-dampened water manometer.

Results

The average fluid removed was 605.63 ± 74.94 mL. Following thoracentesis, 6-MWT, FVC, and FEV1 values increased (p < 0.05), whereas modified Borg dyspnoea scale decreased (p < 0.05). Statistical correlations (p < 0.001) between pleural fluid aspirated and FVC (r = 0.49) and pre-aspiration dyspnoea score were correlated with pre-aspiration pleural pressure with a Spearman’s coefficient of rank correlation (ρ) 0.738 and a p-value of <0.001, suggesting a strong positive correlation and significant correlation.

Conclusion

The correlation between pre-aspiration pleural pressure and dyspnoea score in patients with pleural effusion is likely attributable to the effects of increased pleural pressure on the function of respiratory muscles, including the diaphragm and chest wall muscles, as well as increased stimulation of lung or chest wall mechanoreceptors or both. Following aspiration, the improvement in dyspnoea and effort intolerance is largely because of the reduction in pleural pressure and the subsequent relief of respiratory muscle dysfunction, which is not fully explained by the improvement in lung volumes.

## Introduction

Pleural effusion refers to the pathological accumulation of fluid within the pleural cavity, which can occur because of either excessive fluid production or impaired absorption [1]. Typically, the pleural space contains a minimal volume of fluid, approximately 10 mL on each side [2]. The presence of pleural effusion is inherently abnormal and often signifies an underlying medical condition [3]. It is recognised as the most prevalent manifestation of pleural disease, with aetiologies that encompass a range of cardiopulmonary disorders as well as symptomatic inflammatory or malignant diseases that necessitate prompt evaluation and intervention [4]. The presence of pleural effusion leads to alteration in lung mechanics, affecting the respiratory system’s static and dynamic compliance.

Dyspnoea is the predominant symptom linked to pleural effusion, primarily arising from the distortion of the diaphragm and chest wall during respiration rather than from hypoxemia [5]. The removal of pleural fluid leads to a notable alleviation of symptoms, enabling patients to resume their daily activities. This symptomatic relief is attributed to enhancements in mechanical function rather than alterations in blood gas levels. Research indicates a significant improvement in pulmonary function, particularly in the forced vital capacity (FVC) and forced expiratory volume in the first second (FEV1), following thoracentesis [6]. In many patients, the drainage of pleural fluid can significantly relieve symptoms, even when there is only a marginal enhancement in gas exchange. Dyspnoea may arise from the underlying conditions responsible for the pleural effusion, including intrinsic lung or heart diseases, obstructive endobronchial lesions, or diaphragmatic paralysis, rather than from the effusion itself. The study aims to evaluate the relationship between the six-minute walk test (6-MWT), dyspnoea score, FVC, and FEV1 and pleural pressure before and after thoracentesis.

## Materials and methods

A total of 48 adult patients diagnosed with pleural effusion through clinical and radiological assessments were included in this prospective hospital-based study. Informed consent was obtained from all participants, along with approval from the Institute of Post Graduate Medical Education & Research (IPGME&R) Research Oversight Committee, Kolkata, India (approval no: Inst/IEC/128). Patients and the public were not involved in the design, conduct, reporting, or dissemination plans of this research. The study aims to find the factors that influence effort tolerance in a patient with pleural effusion and the correlating factors, if any.

Patients were enrolled based on the following inclusion criteria: individuals with moderate to massive symptomatic pleural effusion who had not undergone previous aspiration during the current episode of illness attending the Outpatient Department of the Department of Pulmonary Medicine, Institute of Post-Graduate Medical Education and Research and Seth Sukhlal Karnani Memorial Hospital, Kolkata, aged 18 years and older, regardless of sex. The exclusion criteria included patients unable to perform the 6-MWT; those with clinical evidence of cardiac, renal, or liver failure; individuals with locomotor disabilities; severely dyspnoeic patients; and those from whom less than 7 mL/kg of fluid was aspirated. Additionally, patients who refused to undergo post-aspiration spirometry or the 6-MWT were also excluded.

After enrolment based on the inclusion and exclusion criteria and obtaining informed consent, patients were subjected to a six-minute walk test (6-MWT), spirometry, and the modified Borg dyspnoea score. These assessments were conducted both before thoracentesis and after the procedure. The pre-aspiration evaluations, including spirometry and dyspnoea scoring, were performed two to four hours before thoracentesis. Pleural aspiration, along with pleural manometry, was then carried out following the completion of all pre-aspiration assessments. The post-aspiration assessments were conducted approximately 24 hours after the procedure. This timing was chosen to allow stabilization of the respiratory status and to capture meaningful physiological changes following fluid drainage.

Chest radiography

All 48 patients included in the study underwent clinical examination, were diagnosed with pleural effusion, and underwent chest radiography before and after the procedure. Pleural effusion is classified as mild, moderate, or massive based on chest X-ray findings.

Spirometry

Spirometry, including reversibility test, was performed maintaining American Thoracic Society (ATS) recommendation using the Recorders & Medicare Systems Medisoft HypAir Compact+ (Medisoft, Sorinnes, Belgium) spirometer machine. The standardised methods for performing spirometry, as mentioned in the ATS guidelines by ATS/European Respiratory Society (ERS) Task Force: Standardisation of Lung Function Testing [7], was adopted for the present study.

Six-minute walk test (6MWT)

The 6-MWT is a widely used exercise test for assessing respiratory diseases and heart failure. It evaluates the cardiopulmonary response to sub-maximal exercise, reflecting activities of daily living and ideal for understanding thoracentesis’s impact on quality of life [8]. The 6-MWT is simple, safe, and well tolerated, requiring only a 30-m hallway and a pulse oximeter, without needing advanced training or equipment. A 30-m hallway is recommended to minimise frequent turns, which can affect the walking distance. The primary measurement is the six-minute walk distance (6-MWD), but additional data, such as blood oxygen saturation and perceived dyspnoea on exertion, are also gathered. Dyspnoea was assessed at both the beginning and conclusion of each test using the modified Borg dyspnoea scale [9,10]. This features a scale ranging from 0 to 10 accompanied by verbal expressions that reflect progressively increasing levels of sensation intensity.

Thoracentesis and pleural manometry

Thoracentesis was performed using a thoracentesis catheter with a 50-mL syringe attached via a stopcock. The catheter was inserted and advanced along the superior edge of the rib using the standard "walking" technique. As the needle was advanced, aspiration was intermittently attempted by pulling back on the syringe plunger. Once pleural fluid return was confirmed, a water column manometer was connected to the system via a 22G needle to measure pleural pressure before further aspiration.

**Figure 1:**
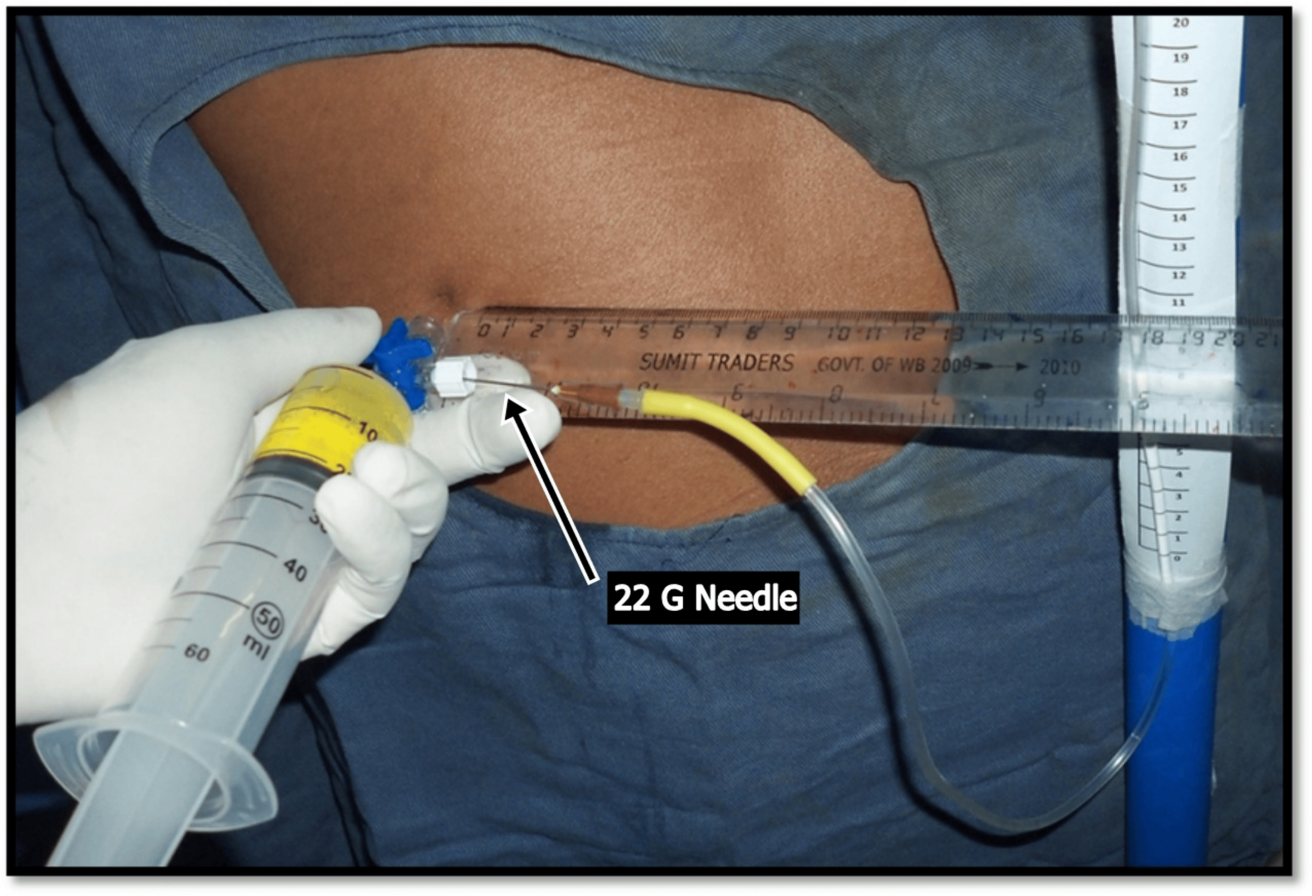
Thoracentesis and Pleural Manometry

Pleural pressure has been measured by an over-damped water column manometer (Figure 1) constructed from two lengths of sterile intravenous tubing pre-filled with normal saline solution and purged of air. A 22G needle is interposed between the thoracentesis equipment and the pleural manometer and serves as a resistor, dampening the pressure oscillations [11]. Without the needle resistor, the water column of a simple water manometer swings widely during respiration and is difficult to record. When using a water manometer, the zero pressure level on the manometer is set at the level that the catheter enters the chest wall. The initial pleural pressure is measured just after insertion of the needle into the pleural space by directly measuring the height of the water column in centimetres from the level of the thoracentesis needle and is approximated to nearest full digit. During subsequent fluid removal, the pleural pressure is measured after each aliquot of 100 mL of pleural fluid was aspirated according to the same procedure and recorded. We stopped fluid removal when the mean pleural pressure decreases to -20 cm H_2_O or when the patient developed coughing, giddiness, or chest discomfort.

Statistical analysis

Data have been summarised by routine descriptive statistics, namely mean and standard deviation (SD) for numerical variables (also median and interquartile range), where these variables are met normally and distributed, and the percentage count for categorical variables is determined. Paired t-test was employed to compare the distribution of categorical variable between subgroups. P < 0.05 considered a significant difference. Pearson’s and Spearman’s correlation coefficient were used for evaluation of correlation between various parameters. All statistical analyses were performed using IBM SPSS Statistics for Windows, Version 26.0 (IBM Corp., Armonk, NY) and Microsoft Office 365 Excel (Microsoft Corporation, Redmond, Washington, USA).

## Results

A total of 48 adult patients with moderate to massive pleural effusion who had no prior history of pleural aspiration were evaluated. Among them, 37 (77%) were men. The mean age of the patients was 37 ± SD 12.6 years; 52% of the cases fell within the 29-48 years age group. Right-sided pleural effusion was observed in 26 (54%) cases, whereas 22 (46%) had left-sided effusion. All cases were classified as exudative effusions. The most common cause of pleural effusion identified in our study was tubercular in origin (68.75%), followed by malignant pleural effusion (22.91%) and infective causes (8.33%).

The average volume of pleural fluid aspirated was 605.63 ± 74.94 mL, with a minimum of 410 mL and a maximum of 720 mL. Aspiration was halted in most patients because of coughing, affecting 27 patients (56.25%). There were no serious adverse effects during or after thoracentesis; no patient exhibited clinical or radiologic signs of pneumothorax or re-expansion pulmonary oedema.

Pre-aspiration spirometry revealed a restrictive ventilatory defect. Following aspiration, the FVC increased by 0.37 SD ± 0.112 L (p < 0.001), whereas the FEV1 increased by 0.23 SD ± 0.11 L (p < 0.001; Table 1). The change in FVC was correlated with the volume of pleural fluid aspirated, yielding a correlation coefficient (r) of 0.49 and a p-value of <0.001, indicating a moderate positive correlation. However, the change in FEV1 did not show any correlation with the amount of fluid aspirated (r = 0.35, p = 0.013).

**Table 1:** Pulmonary Function Results Before and After Thoracentesis FVC: forced vital capacity, FEV1: forced expiratory volume in the first second, FEV1/FVC: forced expiratory volume in the first second/forced vital capacity ratio.

Parameters	Pre-Thoracentesis	Post-Thoracentesis	Change	t-Value	P-Value
FVC					
Litres	2.03 ± SD 0.628	2.40 ± SD 0.636	0.37 ± SD 0.112	22.71	<0.001
%	58.96 ± SD 13.61	70.01 ± SD 14.20	11.053 ± SD 4.02	19.03	<0.001
FEV1					
Litres	1.87 ± SD 0.58	2.10 ± SD 0.56	0.23 ± SD 0.11	14.65	<0.001
%	63.23 ± SD 14.32	71.66 ± SD 14.21	8.43 ± SD 4.57	12.79	<0.001
FEV1/FVC	91.91 ± SD 5.85	87.70 ± SD 5.17	-4.12 ± SD 3.99	-7.3053	<0.001

The average pre-aspiration 6-MWD was 345.21 SD ± 64.54 m, whereas the average post-aspiration 6-MWD was 423.23 SD ± 66.48 m, resulting in an average improvement of 78.02 SD ± 55.20 m post-aspiration. The average pre-aspiration dyspnoea score, as measured by the modified Borg dyspnoea scale, was 5.58 SD ± 1.20, compared to an average post-aspiration dyspnoea score of 3.17 SD ± 0.778, reflecting an average change of 2.75 SD ± 1.19 (p < 0.001) following aspiration. The average pre-aspiration pleural pressure in cm of H_2_O was -0.92 SD ± 4.41, whereas the average post-aspiration pleural pressure was -8.52 SD ± 3.47, indicating an average change of 7.60 SD ± 2.66 after aspiration.

**Figure 2:**
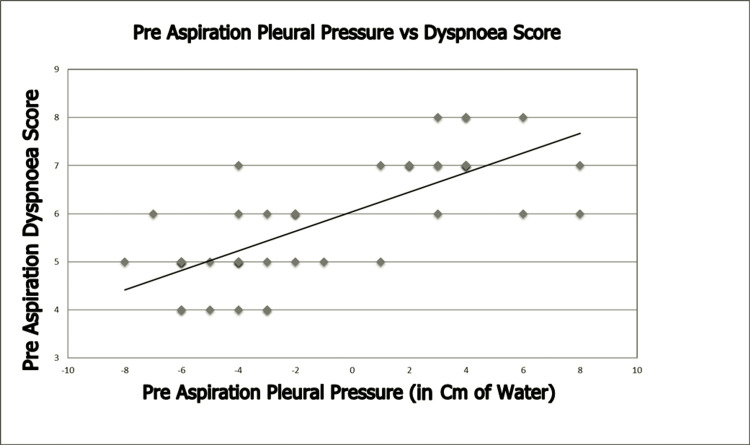
Association Between Pre-Aspiration Pleural Pressure and Modified Borg Dyspnoea Scale Score (ρ = 0.738, p < 0.001)

**Figure 3:**
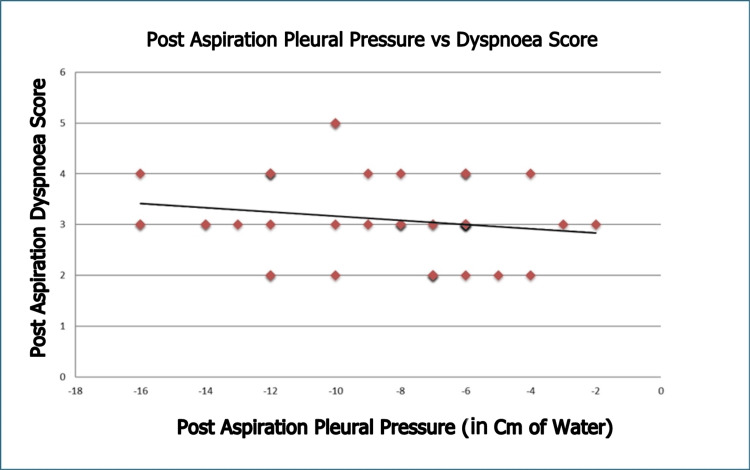
Association Between Post-Aspiration Pleural Pressure and Modified Borg Dyspnoea Scale Score (ρ = -0.176, p = 0.232)

**Figure 4:**
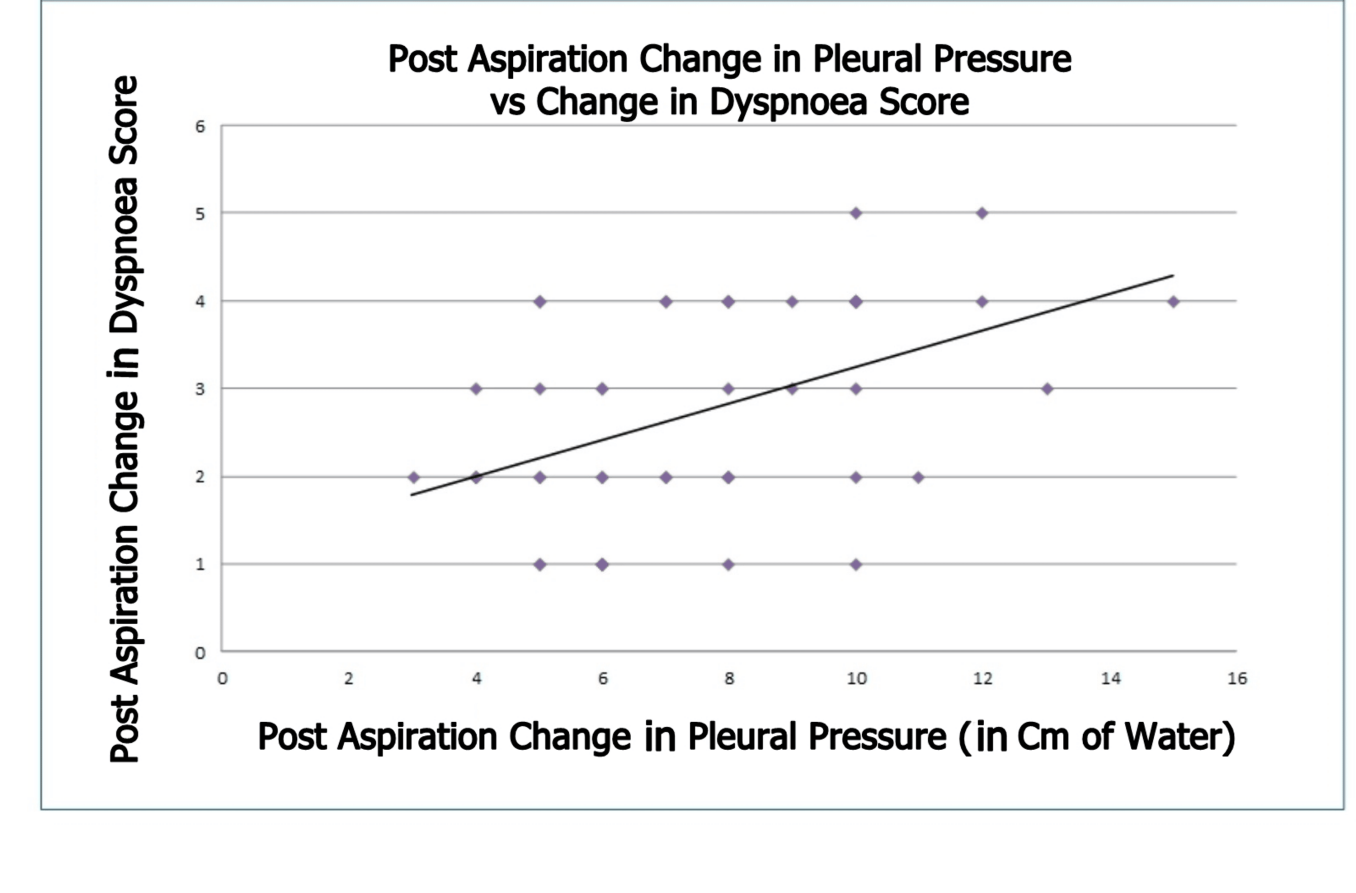
Association Between Change in Pleural Pressure and Change in Modified Borg Dyspnoea Scale Score (ρ = 0.451, p < 0.005)

The pre-aspiration dyspnoea score was significantly correlated with pre-aspiration pleural pressure (ρ = 0.738, p < 0.001; Figure 2), suggesting a strong positive correlation. In contrast, the post-aspiration dyspnoea score did not correlate with post-aspiration pleural pressure (ρ = -0.176, p = 0.232; Figure 3), indicating no correlation. However, the change in post-aspiration dyspnoea score was correlated with the change in pleural pressure (ρ = 0.451, p < 0.005; Figure 4), suggesting a moderate positive correlation.

## Discussion

This study has demonstrated that thoracentesis resulted in a statistically significant change (p < 0.01) in dyspnoea scores as per the 6-MWD (Δ6-MWD = 78.02 ± 55.20 m), modified Borg dyspnoea scale, FVC (ΔFVC = 0.37 ± 0.11 L), and pleural pressure. Previous research has indicated that spirometry variables and the 6-MWT improved following thoracentesis [12-14].

It is important to note that the functional improvement observed at rest may not be applicable to situations involving exertion, even during moderate exercise such as daily activities. Patients with pleural effusions often report a diminished capacity to perform their routine tasks. In this context, improvement in the 6-MWD following aspiration suggests a significant reduction in stress during exercise, which limits the daily activities of patients with pleural effusion. Therefore, thoracentesis provides benefits by enabling patients to return to a more active lifestyle.

Our study revealed that the pre-aspiration dyspnoea score correlated significantly with pre-aspiration pleural pressure (ρ = 0.738, p < 0.001), especially when pleural pressure was above the normal physiological range (-5 to -10 cm H_2_O), most likely by hampering muscle function and diaphragmatic excursion. Estenne et al. [15] show that thoracentesis resulted in only minor changes in pulmonary function and mechanics within two hours after pleural aspiration. These changes are insufficient to account for the immediate improvements in dyspnoea experienced by patients. They observed that thoracentesis consistently led to a shift in the inspiratory pleural pressure-volume curve [16], resulting in markedly more negative pressures generated by the inspiratory muscles at any comparable lung volume, primarily because of a decrease in thoracic volume.

In a study done by Light et al. [17], in 26 patients, vital capacity improved 410 ± 390 mL in patients who had 1,740 ± 900 mL fluid removed. According to the study, the improvement in vital capacity showed the strongest correlation with pleural pressure after the withdrawal of 800 mL of pleural fluid (r = 0.57, p < 0.005). Additionally, the ratio of vital capacity (VC) improvement to the volume of fluid removed correlated most significantly with the change in pleural pressure at the 800 mL mark (r = -0.43, p < 0.05). In line with these findings, our study also demonstrated a moderate positive correlation between the change in FVC and the volume of pleural fluid aspirated (r = 0.49, p < 0.001), suggesting that the amount of fluid removed plays a significant role in improving lung function post-thoracentesis. Patients who experienced smaller decreases in pleural pressure or maintained higher pleural pressures after the removal of 800 mL tended to show greater improvements in pulmonary function following thoracentesis. However, the study did not assess the relationship between pleural pressure and dyspnoea score.

Meglič et al. [18] evaluated pleural pressure measurements during therapeutic thoracentesis and reported that initial pleural pressure was significantly associated with pre-thoracentesis visual analogue scale scores for dyspnoea (ρ = 0.20, p = 0.049) as well as with dyspnoea relief at two hours (ρ = −0.22, p = 0.028) and 24 hours post-procedure (ρ = −0.32, p = 0.006). However, no significant correlation was found between initial pleural pressure and immediate dyspnoea relief following thoracentesis (ρ = −0.15, p = 0.14). The study also demonstrated associations among initial and final pleural pressures (ρ = 0.67, p < 0.001), diaphragm elevation (ρ = 0.44, p < 0.001), and the volume of pleural fluid removed. This study also aligns with our study . This study is consistent with our findings, although the correlation between pre-thoracentesis pleural pressure and pre-thoracentesis dyspnoea scores was stronger in our study.

We propose that the correlation between pre-aspiration pleural pressure and dyspnoea score is likely attributable to the effects of increased pleural pressure, which impairs the function of respiratory muscles, including the diaphragm and chest wall muscles, as well as increased stimulation of lung or chest wall mechanoreceptors, or both, following aspiration.

Although ultrasound (USG) was available in the hospital, it was not accessible for use in this study due to high clinical demand and limited availability of the machine. As a result, thoracentesis was guided by clinical and radiological findings alone. This limitation has been acknowledged.

## Conclusions

Pleural effusion commonly causes dyspnoea because of lung compression and restricted ventilation. Thoracentesis is a standard intervention used to relieve symptoms and improve lung function. While spirometry improvement is documented, the exact role of pleural pressure changes in influencing dyspnoea and exercise capacity remains less clearly defined. This study demonstrates that changes in pleural pressure following thoracentesis correlate significantly with improvements in dyspnoea and 6-MWT performance, especially when pleural pressure is close to normal. It highlights that pleural pressure dynamics, beyond just spirometric changes, play a crucial role in symptom relief. These findings suggest that measuring pleural pressure during thoracentesis could provide valuable clinical insights into patient outcomes.

This study emphasises the clinical relevance of pleural pressure monitoring during thoracentesis. It suggests that integrating pleural pressure measurement with diaphragm movement monitoring via ultrasound may offer a more comprehensive assessment of physiological improvement. This combined approach could guide safer and more effective fluid removal, reducing the risk of complications such as re-expansion pulmonary oedema. It may prompt a shift towards personalised thoracentesis protocols based on pressure dynamics and diaphragmatic function rather than fixed volume targets. These findings could inform future research and support the development of evidence-based procedural guidelines for managing pleural effusion.
